# Optimizing Care Delivery in Patients with Chronic Kidney Disease in the United States: Proceedings of a Multidisciplinary Roundtable Discussion and Literature Review

**DOI:** 10.3390/jcm13051206

**Published:** 2024-02-20

**Authors:** Jamie S. Hirsch, Samuel Colby Danna, Nihar Desai, Ty J. Gluckman, Manisha Jhamb, Kim Newlin, Bob Pellechio, Ahlam Elbedewe, Evan Norfolk

**Affiliations:** 1 Northwell Health, Northwell Health Physician Partners, 100 Community Drive, Floor 2, Great Neck, NY 11021, USA; 2 VA Southeast Louisiana Healthcare System, 2400 Canal Street, New Orleans, LA 70119, USA; 3 Section of Cardiovascular Medicine, Yale School of Medicine, 800 Howard Avenue, Ste 2nd Floor, New Haven, CT 06519, USA; 4 Providence Heart Institute, Center for Cardiovascular Analytics, Research, and Data Science (CARDS), 9205 SW Barnes Road, Suite 598, Portland, OR 97225, USA; 5 Division of Renal-Electrolyte, University of Pittsburgh, 3550 Terrace St., Scaife A915, Pittsburgh, PA 15261, USA; 6 Sutter Health, Sutter Roseville Medical Center, 1 Medical Plaza Drive, Roseville, CA 95661, USA; 7 RWJ Barnabas Health, Cooperman Barnabas Medical Center, 95 Old Short Hills Rd., West Orange, NJ 07052, USA; 8 The Kinetix Group, 29 Broadway 26th Floor, New York, NY 10006, USA; 9 Geisinger Medical Center—Nephrology, 100 North Academy Avenue, Danville, PA 17822, USA

**Keywords:** chronic kidney disease (CKD), care pathways, CKD management, barriers, limitations, recommendations

## Abstract

Background: Approximately 37 million individuals in the United States (US) have chronic kidney disease (CKD). Patients with CKD have a substantial morbidity and mortality, which contributes to a huge economic burden to the healthcare system. A limited number of clinical pathways or defined workflows exist for CKD care delivery in the US, primarily due to a lower prioritization of CKD care within health systems compared with other areas (e.g., cardiovascular disease [CVD], cancer screening). CKD is a public health crisis and by the year 2040, CKD will become the fifth leading cause of years of life lost. It is therefore critical to address these challenges to improve outcomes in patients with CKD. Methods: The CKD Leaders Network conducted a virtual, 3 h, multidisciplinary roundtable discussion with eight subject-matter experts to better understand key factors impacting CKD care delivery and barriers across the US. A premeeting survey identified topics for discussion covering the screening, diagnosis, risk stratification, and management of CKD across the care continuum. Findings from this roundtable are summarized and presented herein. Results: Universal challenges exist across health systems, including a lack of awareness amongst providers and patients, constrained care team bandwidth, inadequate financial incentives for early CKD identification, non-standardized diagnostic classification and triage processes, and non-centralized patient information. Proposed solutions include highlighting immediate and long-term financial implications linked with failure to identify and address at-risk individuals, identifying and managing early-stage CKD, enhancing efforts to support guideline-based education for providers and patients, and capitalizing on next-generation solutions. Conclusions: Payers and other industry stakeholders have opportunities to contribute to optimal CKD care delivery. Beyond addressing the inadequacies that currently exist, actionable tactics can be implemented into clinical practice to improve clinical outcomes in patients at risk for or diagnosed with CKD in the US.

## 1. Introduction

### 1.1. Epidemiology of CKD

Chronic kidney disease (CKD)—defined as abnormalities of kidney structure or function (glomerular filtration rate [GFR] < 60 mL/min/1.73 m^2^ or urine albumin-to-creatinine ratio [uACR] ≥ 30 mg/g) present for at least 3 months—is an underdiagnosed public health crisis [1,2]. The US Centers for Disease Control and Prevention (CDC) estimate that 1 in 7 (14%; ~37 million) adults in the US are living with stage 1 to 4 CKD [3,4]. Unfortunately, because of the asymptomatic nature of early-stage CKD, 9 of 10 adults are unaware of their condition, and 2 in 5 patients with severe CKD are unaware of their disease [4]. CKD contributes to mortality worldwide, and it is estimated that by the year 2040, it will be the fifth leading cause of years of life lost [5]. These unmet needs highlight the need for a review of CKD care delivery and associated barriers in the US and provide the basis for a roundtable discussion of the topic, which are presented here in this article.

### 1.2. Risk Factors for CKD

In the United States, diabetes and hypertension are the leading causes of kidney failure, accounting for three of four new cases [3]. However, it is frequently debated whether hypertension is a cause or effect of kidney disease [6]. Given obesity’s increasing prevalence and status as a risk factor in the development of hypertension and diabetes, it should also be recognized as an indirect cause of kidney failure [7,8]. Other risk factors include cardiovascular disease (CVD), a family history of CKD, inherited kidney disorders, prior history of kidney damage, and older age [4]. An age-related decline in GFR exists and hence, there is a direct correlation between increasing age and prevalence of CKD. According to the National Health and Nutrition Examination Survey (NHANES) and CKD Epidemiology Collaboration (CKD-EPI), CKD is more common in people at least 65 years of age (38%) than those who are 45 to 65 (12%) or 18 to 44 (6%) years of age [4]. CKD is also more common in Black adults (18.8%) than non-Hispanic White adults (13.8%) [3]. People with CKD are at an increased risk for CVD, and CVD represents a leading cause of death in this population regardless of the stage [3]. With increases in the severity of CKD comes a greater risk of CVD, hospitalizations, and associated mortality [9].

### 1.3. Staging of CKD

**Figure 1:**
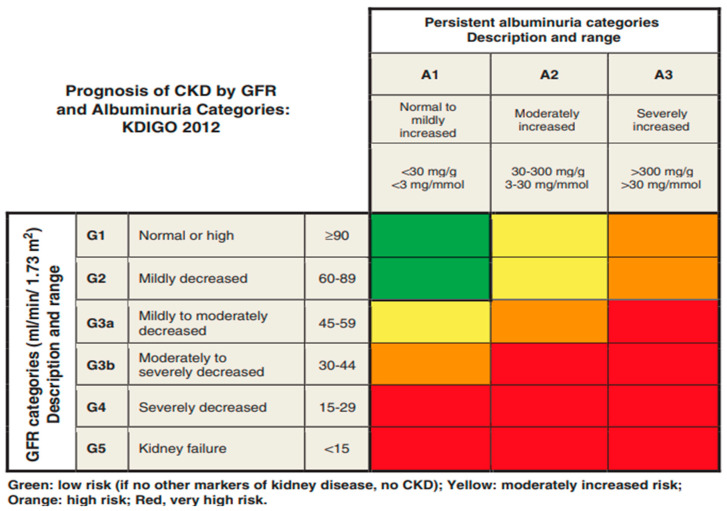
Prognosis of CKD by GFR and albuminuria category (original heatmap currently being revised) [1,10,17,18]. A, albuminuria stage; CKD, chronic kidney disease; G, glomerular stage; GFR, glomerular filtration rate; KDIGO, Kidney Disease: Improving Global Outcomes.

Based on etiology, CKD generally has been categorized by the KDIGO 2014 (original) heatmap, constructed by using estimated GFR (eGFR) as stages 1–5, or less commonly by albuminuria, as stage A1, A2, or A3 (Figure 1) [1,10]. Severity of kidney disease increases with the stage; end-stage renal disease (ESRD) is the most severe presentation and often necessitates kidney replacement therapy (e.g., dialysis) or kidney transplantation for survival [4]. Although most health systems have a wide availability for the use of both serum creatinine and albuminuria in the staging of kidney disease, albuminuria is frequently underassessed [11,12,13,14]. The importance of focusing on the albuminuria component, in addition to the eGFR component, should be recognized, as underutilization has been shown to negatively impact nephrology care [13,14,15]. This is of particular importance for patients undergoing renal cancer surgery since pre-operative proteinuria was found to be an independent predictor of mortality in a systematic review [16].

### 1.4. Economic Implications

Beyond the clinical burden of CKD, the associated economic burden is considerable [19,20,21]. For example, in 2021, the treatment of Medicare beneficiaries with CKD (exclud–ing those with ESRD) cost USD 76.8 billion [3]. A recent healthcare resource utilization analysis demonstrated that, even for early-stage kidney disease, the economic burden is substantial [17]. Therefore, preventing CKD or decreasing CKD progression can alleviate some of the financial burden to the healthcare system.

### 1.5. Clinical Guidelines and Societal Work

Various professional societies have published evidence-based clinical practice guidelines for the screening, diagnosis, evaluation, monitoring, and management of CKD [1,22,23,24,25,26,27]. The guidelines by “Kidney Disease: Improving Global Outcomes” (KDIGO), currently under revision, and the National Kidney Foundation Kidney Disease Outcomes Quality Initiative (NKF/KDOQI) [1,22] are the most widely recognized in the US. “Management of CKD in Primary Care”, from the Veterans Administration and Department of Defense (VA/DoD) [24] provides another clinical practice guideline.

Given the importance of etiologic factors for CKD and the need for appropriate measures to attenuate disease progression, guidelines specific to concomitant kidney disease and hypertension [18] or diabetes [17,25,28] were developed. Additionally, clinical practice guidelines for nutrition in CKD [26] were created, owing to the importance of dietary considerations in patients at risk for or with CKD.

Some society recommendations for CKD include algorithms for screening, evaluation, and/or management [23,24,27]; however, the algorithms are diverse and often cumbersome. In addition, recommendations continue to evolve as our understanding broadens. For example, in a 2014 KDIGO commentary, two tests—the uACR and eGFR—were originally recommended to screen for, diagnose, and manage CKD (Figure 1) [23]. Based on new data, KDIGO guideline updates to the 2014 heatmap are now underway to reflect current knowledge about the care and risk of complications in patients with CKD. However, results from real-world studies of at-risk patients showed that testing is low and/or variable in routine clinical care [29,30].

An increasing multitude of equations to estimate GFR were developed across the past few decades. During this same period, some of these equations became routinely integrated into laboratory reporting systems. A notable advance in the drive toward the race-free diagnosis of kidney disease was also finally outlined by the NKF and American Society of Nephrology (ASN) Task Force on Reassessing the Inclusion of Race in Diagnosing Kidney Diseases. The task force recommended the adoption of a new eGFR 2021 CKD EPI creatinine equation that estimates kidney function without a race variable, as well as increased, routine, and timely use of cystatin C, combined with serum creatinine; this equation is especially to confirm eGFR in adults at risk for or who have CKD [31]. Of note, cystatin C, an alternative glomerular filtration marker, is considered to be a confirmatory test that appears to be more accurate for determining GFR and more sensitive and specific for assessing CVD mortality risks, especially among those with milder disease [32,33].

### 1.6. Treatment Recommendations

**Figure 2:**
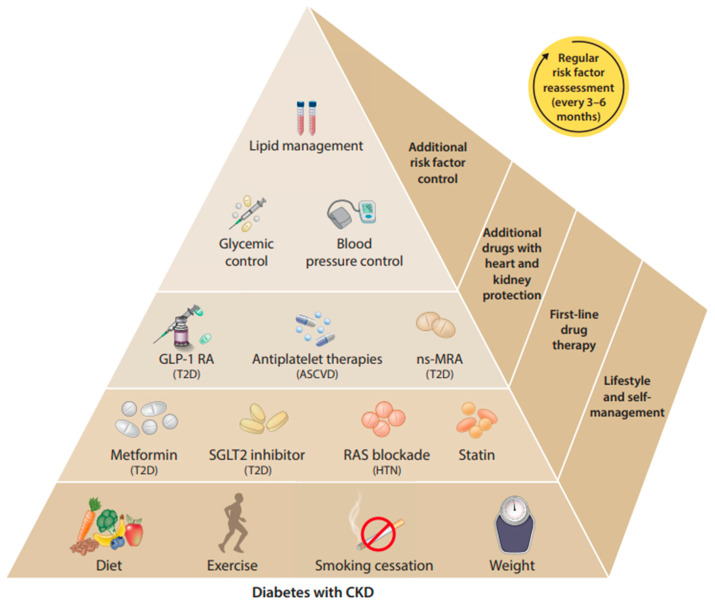
Comprehensive management of patients with CKD and diabetes [24]. ASCVD, atherosclerotic cardiovascular disease; CKD, chronic kidney disease; GLP-1 RA, glucagon-like peptide-1 receptor agonist; HTN, hypertension; ns-MRA, nonsteroidal mineralocorticoid receptor antagonist; RAS, renin–angiotensin system; SGLT2, sodium–glucose cotransporter-2; T2D, type 2 diabetes.

Evidence-based guidelines recommend several management strategies in patients with CKD, especially in those with hypertension or diabetes, including control of blood pressure, diabetes, lipids, and lifestyle modifications to improve kidney and cardiovascular outcomes (Figure 2). Absent patient-specific contraindications, renin–angiotensin system inhibitors (e.g., angiotensin-converting enzyme inhibitors [ACEIs] or angiotensin II-receptor blockers [ARBs]), statins, nonsteroidal mineralocorticoid receptor antagonists (MRAs), and sodium–glucose cotransporter-2 protein inhibitors (SGLT2Is) have all demonstrated benefits in patients with CKD and should be considered for treatment [17,25]. Although beyond the scope of this review, these classes of drugs have been shown either to delay the progression of CKD or attenuate adverse cardiovascular outcomes.

Special consideration should be given to treatment of elderly patients with CKD, who typically have multiple comorbidities and polypharmacy and may be in a frail condition [34]. Because of sarcopenia in the elderly, GFR is generally overestimated when using SCr or uACR and can lead to overdosing of medications. The risk stratification of those likely to progress to ESRD is very important (e.g., whereas younger patients with CKD are more likely to progress vs. older patients demonstrating age-related decline in kidney function and low likelihood of reaching ESRD). Treatment targets need to shift accordingly across the lifespan. In the elderly, the management strategy may need to be modified to a more conservative approach, focusing on quality of life measures rather than aggressively trying to prevent progression to ESRD.

### 1.7. Limitations of Guidelines

Although various clinical practice guidelines for the screening, diagnosis, evaluation, monitoring, and management of CKD exist, provider uncertainty remains. For one, providers may not be fully informed about the guidelines [35]. More concerningly, many clinical practice guidelines are outdated [1,22,24,27], with quality ranging from moderate to high. Recommendations vary; most guidelines cover management of patients with a high risk or advanced CKD as opposed to those with a low risk of CKD progression [36]; consensus about care pathways is limited [37]; and prevention, recognition, and treatment strategies are critically underused [11,38]. For example, at-risk individuals are not being screened during the early stages of disease, when treatment could reduce unfavorable outcomes, such as CVD, dialysis, and transplant, as well as lessen the socioeconomic burden [39,40]. Another possibility is that, despite the recommendation to use both eGFR and uACR to assess kidney damage and function among patients with diabetes, clinicians either selectively adhere to guideline recommendations or only order chemistries with automated reporting, as evidenced by the high use of eGFR testing as compared to the less frequent use of uACR testing [41]. Clinicians and health systems could benefit from a simplified and inclusive process that streamlines delivery of care for patients with CKD, as well as quality of care indicators for at-risk patient populations [42]. Finally, guidelines are not meant to replace the standard of care or to be a “one-size-fits-all” approach but rather assist healthcare providers in making clinical decisions for their patients.

## 2. Overcoming Barriers to CKD Care Delivery

### 2.1. Methodology

The CKD Leaders Network, which was formed in 2020, is a multidisciplinary network of health-system leaders in CKD who came together with the common mission to define and spread a best practice model for population health-driven CKD management. The CKD Leaders Network proposed that a meeting be held to better understand key factors and barriers impacting CKD care delivery in the US. This Program was made possible, by an independent grant from Boehringer Ingelheim Pharmaceuticals, Inc. and Eli Lilly and Company, who provided financial support for the Program. The authors meet criteria for authorship as recommended by the Interna-tional Committee of Medical Journal Editors (ICMJE) and were fully responsible for all aspects of the trial and publication development.

On 17 April 2023, the CKD Leaders Network conducted a virtual, 3 h, roundtable discussion with eight experts including six physicians, one nurse practitioner, and one pharmacist. The participants were chosen based upon their expertise, institutions’ archetype, geography, local prevalence of CKD and comorbidities (e.g., type 2 diabetes, heart failure), eagerness to collaborate, and availability to participate in the roundtable. Additionally, participants filled out contracts prior to the roundtable discussion and they were required to disclose any conflicts of interest.

A pre-meeting survey was conducted with the participants in order to capture individual perceptions relating to CKD care delivery challenges. The findings from the pre-meeting survey informed the meeting facilitation plan, which was used by CKD Leaders Network leadership to ensure an efficient and effective roundtable meeting. The primary topics under discussion were the challenges and key factors affecting clinicians as they screen, diagnose, risk stratify, and manage patients with CKD across the care continuum. Ahlam Elbedewe, a member of the CKD Leaders Network, was the moderator and facilitated the meeting. Objectives of the roundtable discussion were to identify gaps and propose actionable solutions in US-based healthcare delivery systems, so as to improve education initiatives and overall awareness of CKD from the clinician, health system, and patient perspective; prioritize early identification and prevention strategies for patients with CKD; identify and address variations in existing care delivery workflows; discuss ways to improve provider bandwidth, specifically for nephrologists and primary care physicians (PCPs) and advanced practice clinicians (APCs), to permit better CKD care pathways; create better economic/financial incentives for identifying and caring for patients with early-stage CKD; and employ more centralized and streamlined data sharing to provide appropriate treatment for patients with CKD. To address care delivery objectives, the experts were asked to evaluate the existing CKD care delivery infrastructure (e.g., guidelines used within their health system, care team composition, and cross-functional team support); identify challenges to CKD care delivery, including barriers relating to the diagnosis, stratification, management, and monitoring of patients with CKD; and identify and prioritize major gaps faced by clinicians. Experts shared best practice models for CKD care delivery and innovative models of care that have been developed in the US. Herein, the details of the roundtable discussion are summarized, supporting the literature provided, and noted where consensus among participants was lacking and opinions were divided.

### 2.2. CKD Education Unmet Needs

#### 2.2.1. Patient and Provider CKD Education Gaps

Various CKD educational resources, covering a wide range of topics, are available to clinicians from myriad professional societies, government bodies, and advocacy groups (Table 1). Despite these resources, the experts agreed that patients do not receive sufficient education about CKD and their diagnosis, which leads to a lack of accountability and understanding during the CKD care journey [43,44]. Patients with early-stage CKD need more education on modifiable factors they can control, such as dietary changes, as well as education regarding social determinants of health, public health areas that are undervalued and need more attention [45]. However, given many older patients with early-stage CKD with multiple comorbidities will not reach higher-stage CKD or ESRD, and the majority being in stage 3a or 3b, education (and interventions) need to be individualized according to the patient’s health status and risk of progression [46]. For example, one study found that healthcare professionals felt that discussions surrounding kidney disease created anxiety in older patients with stage 3a kidney disease where the benefit of education/intervention might be deemed less effective [47]. Therefore, data suggest that consideration should be given to the entirety of the population of CKD and what types of education are necessary for the clinical situation and vehicles (e.g., technology) used to deliver that information since all patients may not have access.

Patients do not always appreciate the importance of certain clinical markers (e.g., blood pressure, low-density lipoprotein (LDL) cholesterol, eGFR, creatinine, uACR, hemoglobin A1c, albuminuria) in CKD and overall health. Unfortunately, adequate education can be time consuming, especially with limited provider availability. Patient education, therefore, is often off-loaded to dedicated educators or pharmacists, who may similarly not have the capacity to provide support. Real-world data about the value of patient education are lacking, and education services pertaining to CKD generally are not well reimbursed. Of importance, patient education about early CKD likely would lead to substantial future health benefits and savings, while payers may be more focused on short-term benefits. Finally, reimbursement challenges (see Resource and Education Reimbursement Challenges) lead to a dearth of support for initiatives to improve patient education.

With respect to resources for providers, the experts agreed that CKD education within health systems is limited, existing resources are not well defined, and knowledge-sharing amongst clinicians could be an important part of education [62]. In other disease states, such as diabetes, educational programs used by providers often are available across the patient spectrum, regardless of disease duration or severity. In contrast, CKD education—similar to clinical practice guidelines—primarily focuses on advanced disease stages.

#### 2.2.2. Resource and Education Reimbursement Challenges

The experts concurred that securing payer reimbursement for education remains difficult. Proving the value of CKD education to payers is therefore of vital importance. Payers must be able to envision future, even eminent, savings and cost benefits. Currently, Centers for Medicare and Medicaid Services (CMSs) only reimburse stage IV CKD education, but that is too late, especially considering preventive measures that could help delay the progression of CKD and minimize costs. Unfortunately, cost/benefit data across the spectrum of CKD over time are lacking.

The experts echoed existing sentiments that a “one-size-fits-all” approach to education is not appropriate [63,64]. Different populations often require different modes of education. Whereas much education is now delivered online, Medicare populations often prefer paper-based educational resources, and they may not always have easy access to electronic materials. In addition, educational materials must be available in multiple languages to reach a wider range of patients from varying demographic backgrounds; in addition, patient-facing education materials must be innovative and current.

#### 2.2.3. Stakeholder Opportunities 

Stakeholders can (1) help educate at-risk individuals, patients with early-stage CKD, and those with later-stage disease with general (e.g., CKD signs and symptoms, assessment, diagnosis, prognosis, testing and treatments) and specific (e.g., modifiable risk and lifestyle factors, what to expect with treatment) information; (2) ensure education is audience-specific, concise, and easy to understand; and (3) offer education across multiple platforms and languages [65]. Entities such as life sciences organizations and professional societies can assist in developing concise, relevant, and current CKD education, as well as patient-facing resources, for clinicians. Programs and content from professional societies (e.g., American Diabetes Association [ADA], NKF) are likely to be better received than information from a pharmaceutical company.

In addition, content developers need to be cognizant of clinicians’ time, bandwidth, and competing priorities, which may impact the ability of care teams to remain current on the latest education. Content developers should leverage digital technology [66], for example, to communicate the patient’s place in their care journey, potential treatment options, and a corresponding path based on the chosen treatment option. Stakeholders should also establish the value of CKD education (e.g., disease progression, quality of life, direct and indirect costs) for payers, so that education can be reimbursed.

### 2.3. Challenges to CKD Care Delivery

Various CKD guidelines are available to clinicians, most of which offer recommendations for identifying, diagnosing, and managing CKD; however, the guidelines have obvious limitations, as mentioned previously, and do not address some practical aspects of CKD care delivery.

#### 2.3.1. Barriers to Identification and Diagnosis of Patients with CKD 

**Figure 3:**
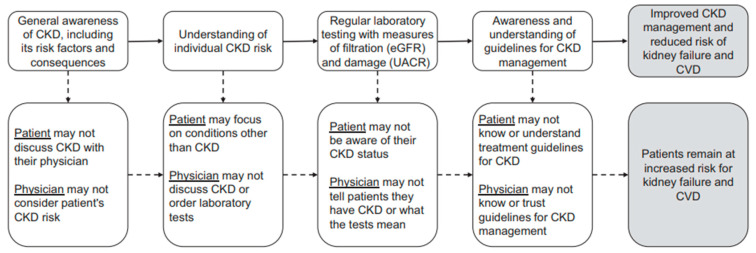
Patient and provider barriers to delivery of optimal CKD care [69]. CKD, chronic kidney disease; CVD, cardiovascular disease; eGFR, estimated glomerular filtration rate; uACR, urinary albumin-to-creatinine ratio.

Multiple barriers to optimal CKD care exist (Figure 3), including low awareness of patients and clinicians of the risk factors and complications of CKD, as well as patient denial or limited involvement in their disease. In some cases, there may be suboptimal clinician knowledge to confirm the CKD diagnosis and conduct appropriate monitoring of CKD (e.g., low use of screening labs, such as serum creatinine at regular intervals; only 1 in 5 patients have adequate uACR monitoring). This lack of knowledge can hinder the diagnosis of early CKD, as well as an appropriate risk stratification of patients. Other barriers include the limited involvement of primary care in management of CKD, a lack of proper nephrology consults based on target GFR and lack of clinician- or patient-targeted evidence-based interventions to reduce CKD progression or CVD, the existence of few partnerships between a clinician and patient, and insufficient tools for utilizing electronic health records (EHRs) [11,12,67,68,69].

Experts agreed that eGFR is viewed as “the bread and butter” of CKD progression measurement because it is “dramatically better than just eyeballing creatine” and provides a standard measurement for identifying CKD [33,70]. Several variations of this measurement have matured with time; however, a main drawback of eGFR remains in that it serves only as a step in identifying CKD; it does not trigger subsequent steps for patients to receive care. Experts also discussed the Kidney Failure Risk Equation (KFRE), another useful tool for CKD risk stratification and prediction [71]. A noted drawback, however, is its reliance on urine albumin, which is missing in up to 70% of patients with CKD [72], although formulas to convert dipstick urine protein or the urine protein-to-creatinine ratio from uACR exist [73].

Reliance on billing codes to identify patients with CKD may be inadequate and may significantly underdiagnose patients with CKD [74]. More than 70% of Medicare beneficiaries with laboratory results suggestive of CKD lacked a diagnosis code for CKD, with one-half of those with stage 3 CKD lacking a diagnosis and White patients less likely to be diagnosed than Black patients [74].

#### 2.3.2. Lack of Bandwidth and Support for Providers

Clinicians undoubtedly lack sufficient time, bandwidth, and/or staffing resources to optimally identify patients across the CKD spectrum or to manage patients throughout the care continuum. As medical knowledge increases and new therapeutic options become available, any changes in disease management approaches that impact a demographic group mainly seen in primary care will greatly impact the workload of primary care physicians. Whereas many nephrologists have the capacity to only treat later-stage CKD, the burden to treat patients with early-stage disease cannot and should not fall exclusively to primary care or general care practitioners, both of which also lack bandwidth.

The experts agreed that, for several reasons, including clinician bandwidth, a lack of patient self-referrals, a lack of patient and clinician education, and other challenges, patients will often reach stage 4 or 5 CKD before they are able to see a nephrologist. Experts noted that time, bandwidth, and competing priorities are also limiting factors for ensuring that care teams remain current on the latest education. Routine monitoring of patients with CKD can be time consuming, however, additional care team members such as advanced practice practitioners and designated educators can play an important supporting role in the management of these patients [75].

#### 2.3.3. Financial Challenges

In addition to lacking sufficient time, bandwidth, and/or staffing resources, experts noted a great need to create enhanced economic incentives for identifying and caring for patients with early-stage CKD. Because of financial incentives associated with later-stage care (e.g., kidney replacement therapy, transplantation), leadership across health systems has traditionally focused on stage 4 and stage 5 CKD. Most health systems continue to use a fee-for-service model, which directly impacts the resources and time dedicated to the delivery of CKD care, because a financial incentive associated with early-stage CKD care is uncommon. Some movement toward value-based care for CKD has taken place, thereby aligning payer and provider interests [76]; however, more data are needed to demonstrate cost savings of value-based contracts in CKD care. Payers must be involved in these conversations and participate in decisions to highlight how preventive CKD measures, especially for patients with diabetes and/or hypertension, can decrease overall costs and lead to improved patient outcomes [76]. Although data on newer medications (such as SGLT2 inhibitors, GLP-1 RA, and ns-MRA) look promising, the long-term cost–benefit analysis of these medications has not yet been determined. Additional research exploring the cost-effectiveness of these diverse groups of patients with CKD is needed.

#### 2.3.4. Stakeholder Opportunities

Stakeholders can (1) standardize diagnostic procedures; (2) provide necessary care team support by creating partnerships with payers and other industry stakeholders; (3) develop and implement technology to automate processes where applicable; (4) create enhanced economic incentives for identifying and caring for patients with early-stage CKD; and (5) demonstrate the cost savings of value-based contracts in CKD care and increase the number of health systems that embrace value-based care.

### 2.4. Existing Care Pathways

Care pathways assist clinicians in providing evidence-based healthcare with the goal of optimizing outcomes. Generally guided by clinical practice recommendations, numerous existing care pathways for CKD exist, but they are diverse in design, content, and implementation [37]. Each is unique, but usually encompasses online interactive tools, CKD screening/diagnosis algorithms, drug management, lifestyle management, nephrology referrals, dissemination plans, a description of target end-users, an evaluation of pathways, cost analyses, and clinical practice guidelines [37].

One online CKD clinical pathway found the interaction time between the user and CKD pathway website to be only 2 min 8 s, and the end-user (provider or researcher) was not clearly defined [77], whereas another resulted in improvements in uACR testing, although primarily in patients who did not have diabetes [78]. Other clinical pathways have been used to support future patient-centered approaches [79], visualize patterns of the co-progression of multiple clinical events in a chronic disease (such as CKD) by using patients’ own clinical data [80], or improve and/or maintain quality of care while reducing costs for patients with CKD (i.e., CMS ESRD treatment choice [ETC] model) [81]. A large systematic review found that several key features led to successful implementation, including framing CKD interventions within the context of cardiovascular health and diabetes, interventions that were considered compatible with existing practices or everyday lives of patients and ownership in the process to allow individualized improvements [82].

#### 2.4.1. Clinical Biomarkers and Risk Prediction Equations

During the past few decades, numerous approaches for estimating GFR have been used in clinical practice [31]. These prediction equations use the biomarkers creatinine or cystatin C (or a combination of both), along with other markers, such as age, sex, race, and other population-specific factors, to estimate kidney function [31]. The earliest “bedside calculation” using creatinine, age, and weight to calculate kidney function and provide a rough estimate of GFR is the Cockcroft–Gault formula [83]. The most widely accepted formulas to calculate GFR have been the CKD-EPI cystatin C 2012 equation [84], the revised bedside Schwartz formula (ages 1–17) [85], the CDK-EPI creatinine–cystatin C 2021 equation [72], and the CKD-EPI creatinine 2021 equation [86].

The NKF recently endorsed the 2021 refit CKD-EPI equation for eGFR using creatinine, age, and sex, without a race coefficient [31,86]. This equation has been shown to be more accurate than other, previously used equations, while avoiding a disproportionate impact on any ethnic group that could lead to differences in the diagnosis and treatment. The standardized cystatin C assay seems to be a better marker than creatinine; it is considered a “confirmatory test” for decreased eGFR and is indicative of an adverse prognosis in CKD [86]. Additionally, combining filtration markers (creatinine and cystatin C) improves accuracy and supports better clinical decisions compared to either marker alone; however, research on eGFR methods with these new endogenous filtration methods is still needed [31]. The added benefits of incorporating cystatin C should be considered according to the patient health status since evidence that the incorporation of this marker leads to an added benefit on relevant clinical end points in a real-world setting is lacking. To support those efforts, the CKD Biomarkers Consortium is currently investigating the discovery and validation of novel biomarkers for CKD to advance research into the causes and outcomes of the disease [87].

Other “calculators” are available that predict the probability of the disease stage, progression, or risk of hospitalization in patients with CKD [71,88,89]. Among adults unaware of possessing CKD, the Screening for Occult Renal Disease (SCORED) tool, which incorporates age, sex, and seven comorbidities (including hypertension, diabetes, and CVD), estimates the probability of having stage 3–5 CKD [57,88]. The Tangri KFRE predicts the probability of CKD progression to ESRD in the next 2 or 5 years by using either a four- or eight-variable equation [57,71]; however, a study found that up to one-half of patients with high-risk CKD were not referred to a nephrologist within a year of the established risk [90]. Lastly, the uACR can help to identify patients with CKD at risk of hospitalization for heart failure [89]. These predictive equations can assist in the identification of patients that should seek more comprehensive medical advice, further medical evaluation, aid in earlier diagnosis and management, as well as slow disease progression. These probabilities could also be used to foster patient and clinician communication, heighten awareness, facilitate appropriate nephrology referrals, and guide optimal disease management for best outcomes [90,91].

#### 2.4.2. Screening and Early Identification of Patients

The experts asserted that the early identification of CKD and implementation of prevention strategies need to be prioritized [92,93]. Identifying patients early in the disease process is an essential step to optimal CKD care delivery [94]. The experts discussed ways that early identification can be achieved. First, upstream interventions and prevention strategies among at-risk populations, such as optimized hypertension and diabetes management, and the evaluation of social determinants of health (e.g., access to healthy foods and safe places to be active), need to be implemented [95]. In addition, the classification of patients needs to be improved (e.g., using new formulas that are not race-based to estimate GFR) [31,86], so that an efficient system of prioritization and triage can be created. To that end, a strong system to identify patients—using dashboards, patient registries, or EHRs, for example—should be in place [96]. Registries can also help identify modifiable care gaps across the spectrum of CKD care and enable the implementation of population health strategies [97]; however, many health systems lack system-wide methods to examine the overall patient population.

Although suggestions have been made, the best method to improve the identification of patients with early-stage CKD is unknown. The first step for an optimal patient journey is identifying those at the greatest risk, perhaps by using an early CKD identification registry or qualification list that is uncapped [98,99]. Purely building off existing lists or registries (such as the National Kidney Foundation Patient Network or institutional EHRs) is not straightforward and likely will be more time consuming than focusing on the highest-risk patients (i.e., cardiorenal–metabolic-type patients, characterized by a combination of type 2 diabetes, hypertension, hyperlipidemia, CKD, and heart failure). While protocols may exist for these patients, they are typically not widely applied across health systems.

#### 2.4.3. Workflows and Referral Pathways

Consensus was reached amongst experts that existing workflows and referral pathways are, for the most part, not standardized or defined within health systems. Tools being used include alerts from laboratories, best practice advisories or other EHR-based decision support, e-consults with primary care, and centers of excellence. In contrast with other disease areas, CKD does not have a set care pathway. CKD care often is initiated by primary care, with the PCP referring patients to a nephrologist. And, as with guideline recommendations and education, existing care pathways often center on the patients with the highest-risk CKD in the later disease stages.

#### 2.4.4. Stakeholder Opportunities

Stakeholders can and should (1) encourage and advocate for the early identification of CKD; (2) standardize workflows and referral pathways within health systems to create a unified CKD care pathway, covering the full spectrum of CKD; (3) establish procedures to triage patients with CKD to create a strong foundation for CKD care delivery; and (4) standardize use of clinical markers and risk-prediction methods.

### 2.5. Optimal CKD Care Delivery

**Figure 4:**
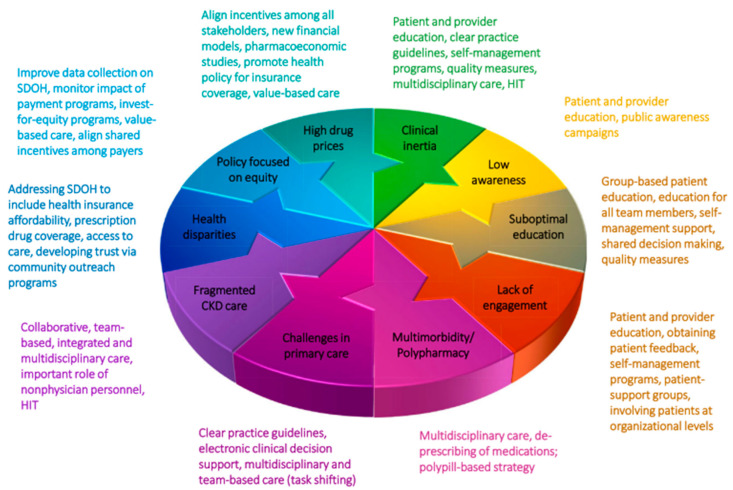
Barriers and strategies in implementing therapies for CKD [76]. CKD, chronic kidney disease; HIT, health information technology; SDOH, social determinants of health.

Successful management of patients with CKD depends on a framework that considers the needs of all patients across the spectrum of disease, from screening to ESRD [100]. If properly implemented, optimal CKD care delivery should follow a model that is patient-centered and individually tailored, of high quality, and low-cost, with goals of early identification, appropriate treatment, and the minimization of disease progression, lessening the burden of CKD, and improving patient outcomes [100,101]. A strategic, comprehensive approach that takes into account barriers to implementation, outlined in Figure 4, should be considered (Figure 4) [102]. Examples of how to optimize CKD care delivery are provided in Table 2.

#### 2.5.1. Characteristics of an Optimal Care Pathway

Effective management of patients with CKD cannot be delivered by nephrologists alone and requires care delivery that includes partnerships with PCPs and other care team members [103]. The Renal Physicians Association (RPA) provided guidance on the delivery of an optimal CKD care pathway to enhance cost-effective, comprehensive kidney care within a value-based system [75]. Key attributes of an optimal care pathway would involve a multidisciplinary CKD clinic structure (with primary care, nephrology, laboratory, administrative, and health equity leadership), a CKD patient care navigator, population health management, a CKD registry, clinical decision support tools, a structured education program, the incorporation of information technology tools, psychosocial support, chronic care management, defined data metrics for improving the pathway, payment model incentives, and Current Procedural Terminology (CPT) coding guidelines and management [75,101]. Government programs, such as the CMS ESRD treatment choice (ETC), Kidney Care First (KCF), and Comprehensive Kidney Care Contracting (CKCC) and Kidney Care Choices (KCC) models, encourage increased use of home dialysis and kidney transplants, as well as the evaluation of new Medicare payment options, to improve quality of care for a patient’s kidney disease, while reducing Medicare expenditures [81,104,105].

#### 2.5.2. Potential Barriers and Limitations

The experts identified multiple barriers and limitations that prevent optimal CKD care delivery. One important challenge is coordinating care when patients are seen by multiple clinicians (e.g., PCPs, cardiologists, nephrologists), across multiple health systems, which can result in ambiguity around new treatment options. For example, if a patient with CKD is seeing multiple clinicians, it can be difficult to identify the reasons for which certain patients receive a particular medication and others do not. Adding to the complexity of these situations is payment for treatment, which remains an important piece of the puzzle. The inclusion of extended care team members in the process, such as pharmacists, financial counselors, and dedicated educators, offers transparency around the costs of different treatment options.

#### 2.5.3. Reimagining CKD Management

First and foremost, there is a need to shift from a traditional fee-for-service model to a value-based approach in the management of patients with CKD. According to the experts, next-generation solutions, such as real-world data generation and artificial intelligence approaches, that enable more centralized and streamlined data sharing are vital to enhancing the identification of at-risk individuals and to improving management of patients with CKD [106,107,108,109]. Owing to their focus on shared data, integrated care networks will be central contributors to creating solutions.

The experts also stressed that patient-level data must be centralized, enabling effective communication among key clinical stakeholders regardless of their location, for coordinated care, and it must be longitudinal for successful long-term disease management. Although centralized patient data can be used to improve care, alternative options should also be available as patients should be given the ability to opt out and limit use of their personal and health data per privacy laws. A centralized data system also requires the use of technology to improve the health of patients with CKD without increasing clinician burdens. For example, automated e-consults and EHR alerts, which are already in use by some health systems and were especially enhanced during the COVID-19 era, can guide clinicians in identifying and referring appropriate patients for further evaluation and management, as well as help to detect and lower risks, decrease hospitalizations, improve outcomes, and forecast adverse events [110,111]. In addition, artificial intelligence has the potential to improve the identification of high-priority patients by creating specific, tailored care delivery algorithms. Furthermore, when deciding on the use of new technologies to improve care, provisions should be taken to accommodate patient groups with low literacy in the use of these technologies.

Machine learning approaches have already been used successfully as a time- and cost-saving method to improve diagnostic screening of CKD, closely monitor patients at risk for CKD so as to identify potential onset earlier, predict risk of ESRD, and determine mortality and treatment of patients with CKD [112,113,114,115]. A model that uses routinely collected laboratory measurements, which are rapidly accessed and can be broadly applied across all stages of CKD, has been suggested as the most optimal [116].

With regards to management, evidence-based practices exist; however, dissemination and implementation science (DIS) is necessary to bridge the gap between research and practice to improve care, process, and outcomes [117]. The ongoing Kidney Coordinated Health Management Partnership (Kidney CHAMP) trial will evaluate the effectiveness for slowing progression to ESRD of a centralized EHR-delivered population health management strategy in high-risk patients with CKD [118]. In this NIH-funded trial (PI Jhamb), the nephrology co-management approach with PCPs provides an efficient way of delivering specialty service to an ever-growing CKD population, especially given the shortage of nephrologists.

#### 2.5.4. Stakeholder Opportunities

Stakeholders can help to develop, provide, and support next-generation solutions by implementing artificial intelligence and machine learning tools in CKD care delivery to negate some existing challenges and barriers to optimal care. In order for artificial intelligence and machine learning to be successful, the views and interests of patients should also be considered and in some cases, patients need to take an active role. The optimal integration of these tools into population health initiatives remains as an area of active investigation, with best practices not fully defined. Additional opportunities exist for stakeholders to generate real-world data and provide evidence of cost savings using a value-based approach.

## 3. Conclusions

In the US, CKD represents a major clinical and economic burden to the healthcare system. Unfortunately, persisting barriers now hamper screening and early diagnoses, adherence to evidence-based guidelines/CKD care pathways, appropriate patient and clinician education, and the implementation of established therapies that can prevent the progression of CKD. However, even given the complexity of multidisciplinary team involvement in CKD care pathways, opportunities do exist to improve outcomes in patients with CKD in a budget-mindful manner. In the future, the use of artificial intelligence and machine learning approaches to help in the screening, identification, and improved management of patients at risk for or previously diagnosed with CKD may provide great benefits for primary care, kidney care, and patients seeking their clinical services.

## Figures and Tables

**Table 1:** Provider-Facing Resources and Tools.

Resource	User(s)	Description	Reference
KART 2.0	Primary care	Validated tool to assess patients’ knowledge of CKD and kidney transplantation	Waterman, A.D.; Nair, D.; Purnajo, I.; Cavanaugh, K.L.; Mittman, B.S.; Peipert, J.D. The Knowledge Assessment of Renal Transplantation (KART) 2.0: Development and validation of CKD and transplant knowledge scales. *Clin. J. Am. Soc. Nephrol*. 2022, *17*, 555–564. [48]
Chronic Kidney Disease Disparities: Educational Guide for Primary Care (CMS)	Primary care	Educational guide Identification of CKD Treating and monitoring of CKD Providing patient-centered care for CKD	https://www.cms.gov/files/document/chronic-kidney-disease-disparities-educational-guide-primary-care.pdf. (accessed on 24 January 2024) [49]
Explaining Your Kidney Test Results: A Tool for Clinical Use (NIDDK, NIH)	CKD care teams and patients	Online and printable tool for clinical use to explain kidney test results in a digestible and understandable way to patients	https://www.niddk.nih.gov/health-information/professionals/advanced-search/explain-kidney-test-results. (accessed on 24 January 2024) [50]
The Kidney Failure Risk Equation Calculator (CKD Network)	CKD care teams and patients	Validated online tool that uses 4- and 8-variable equations to accurately predict the 2- and 5-year probability of treated kidney failure (dialysis or transplantation) for a potential patient with stage 3 to 5 CKD	https://kidneyfailurerisk.com. (accessed on 24 January 2024) [51]
eGFR Calculator (NKF)	CKD care teams	App for estimating kidney function that has five sperate eGFR calculators: CKD-EPI creatinine 2021 equation (preferred method) Revised bedside Schwartz formula (for ages 1–17) Cockcroft–Gault formula CKD-EPI cystatin C 2012 equation CKD-EPI creatinine–cystatin C 2021 equation	https://www.kidney.org/apps/professionals/egfr-calculator (accessed on 24 January 2024) [52]
Kidney Disease for Health Professionals (NIDDK)	CKD care teams	Online resource for information about Identifying and managing patients Laboratory evaluations Talking with patients about kidney disease	https://www.niddk.nih.gov/health-information/professionals/clinical-tools-patient-management/kidney-disease (accessed on 24 January 2024) [53]
Relative Risk, Monitoring and Referral in Patients with CKD (NKF)	CKD care teams	App that summarizes new science to explain how eGFR and uACR are independent risk factors for ○ All-cause mortality ○ Cardiovascular mortality ○ Kidney failure ○ Acute kidney injury ○ Progressive CKD App suggests monitoring frequency and referral decision, depending on categories of eGFR and uACR App provides rapid, convenient learning at your fingertips	https://www.kidney.org/apps/professionals/relative-risk-monitoring-and-referral-patients-ckd (accessed on 24 January 2024) [54]
KDOQI App (NKF)	CKD care teams	App that can be used to access guidelines and commentaries	https://www.kidney.org/apps/professionals/kdoqi-app (accessed on 24 January 2024) [55]
United States Renal Data System (NIDDK)	Medical researchers and CKD care teams	Online, national data system that collects, analyzes, and distributes information about CKD and ESRD in the United States	https://www.niddk.nih.gov/about-niddk/strategic-plans-reports/usrds (accessed on 24 January 2024) [56]
Chronic Kidney Disease Risk Calculators (CDC)	Medical researchers and CKD care teams	Online risk calculators for Risk of having stage 3 to 5 CKD Risk of progression of CKD	https://nccd.cdc.gov/ckd/Calculators.aspx (accessed on 24 January 2024) [57]
Medication Therapy Management	CKD care teams	Programs for clinicians and patients for medication management/self-care management	https://doi.org/10.2215/CJN.06790617 (accessed on 24 January 2024) [58] https://doi.org/10.1053/j.ackd.2014.02.011 (accessed on 24 January 2024) [59] https://doi.org/10.1053/j.ajkd.2021.05.023 (accessed on 24 January 2024) [60] https://doi.org/10.1186/s12882-016-0279-6 (accessed on 24 January 2024) [61]

ASN, American Society of Nephrology; CDC, Centers for Disease Control and Prevention; CKD, chronic kidney disease; CMS, Centers for Medicare and Medicaid Services; eGFR, estimated glomerular filtration rate; EPI, Epidemiology Collaboration; ESRD, end-stage renal disease; KART, Knowledge Assessment of Renal Transplantation; KDOQI, Kidney Disease Outcomes Quality Initiative; NIDDK, National Institute of Diabetes and Digestive and Kidney Diseases; NIH, National Institutes of Health; NKF, National Kidney Foundation; uACR, urinary albumin-to-creatinine ratio.

**Table 2:** Real-world Examples from Roundtable Participants and Implementation of Optimizing CKD Care and Delivery.

Category	Example	Implementation into Routine Care
Multidisciplinary collaboration		
“At Ochsner, primary care groups meet for weekly care huddles, sharing clinical information regularly to exchange ways to best care for their patients.”		Conduct regularly scheduled multidisciplinary meetings
“University of Pittsburgh Medical Center [UPMC] has raised the priority of CKD within the leadership of the health system by undergoing a partnership to improve CKD care from a population health perspective. The approach has included a large, multidisciplinary care team to manage patients, including nephrologists, pharmacists, PCPs, educators, social workers, dieticians, etc.”		Ensure all stakeholders are included in CKD population health management
“There are numerous health systems within major cities; therefore, it is not uncommon for patients to see multiple doctors within multiple health systems. This often leads to important patient information not being shared across systems, and as a result, gaps in care.”		Develop a method/database to ensure sharing of clinical information, including EHR interoperability, with easy access for all providers in the health system
Diagnosis and Management of CKD		
“The nephrologists at Yale don’t have the bandwidth to see patients that have an eGFR of <80; typically, the referral to the nephrologist doesn’t happen until a patient is on or nearing dialysis.”		Education of primary care providers for appropriate CKD management strategies
“By intervening in the early stages of CKD, we can reduce the number of patients who go on dialysis—this would be a major cost-saving opportunity.”		Earlier identification of CKD and initiation of appropriate therapeutic strategies to slow or prevent progression of CKD
“Our health system currently estimates a patient’s GFR; however, there is an opportunity to better integrate it into population health efforts to support downstream interventions.” “UPMC has also implemented the Tangri KFRE to create a risk profile. And, once they’ve identified the highest-risk patients, they automatically enroll them into the program, where the PCPs get an e-consult, and the patient receives all the ancillary services.”		Develop institutional algorithm for CKD identification and risk assessment to trigger appropriate consultations
“At Geisinger, there is a referral alert in place to refer a patient to a nephrologist when they are diagnosed with stage 4 CKD, but the alert is not put in at earlier stages of CKD. The alert was put in place to triage later-stage CKD, as the nephrologist does not have the ability to see all patients with CKD in the health system.”		Set up automatic alerts for referrals at clinical threshold, but also educate primary care provider for CKD management strategies
Other		
“UPMC has built out a CKD registry in Epic, which currently contains approximately 80,000 patients across CKD stages 3, 4, and 5.”		Develop institution registry and create awareness so that patients can be appropriately managed
“Dialysis patients have now become eligible for Medicare Advantage plans.”		Ensure enrollment into Medicare Advantage for eligible patients
